# Fusion of Multimodal Imaging and 3D Digitization Using Photogrammetry

**DOI:** 10.3390/s24072290

**Published:** 2024-04-03

**Authors:** Roland Ramm, Pedro de Dios Cruz, Stefan Heist, Peter Kühmstedt, Gunther Notni

**Affiliations:** 1 Fraunhofer Institute for Applied Optics and Precision Engineering IOF, Albert-Einstein-Str. 7, 07745 Jena, Germany; 2 Faculty of Mechanical Engineering, Technical University Ilmenau, Ehrenbergstraße 29, 98693 Ilmenau, Germany

**Keywords:** 3D digitization, multi-sensor systems, multimodal image fusion, photogrammetry, structure from motion, multimodal, multispectral

## Abstract

Multimodal sensors capture and integrate diverse characteristics of a scene to maximize information gain. In optics, this may involve capturing intensity in specific spectra or polarization states to determine factors such as material properties or an individual’s health conditions. Combining multimodal camera data with shape data from 3D sensors is a challenging issue. Multimodal cameras, e.g., hyperspectral cameras, or cameras outside the visible light spectrum, e.g., thermal cameras, lack strongly in terms of resolution and image quality compared with state-of-the-art photo cameras. In this article, a new method is demonstrated to superimpose multimodal image data onto a 3D model created by multi-view photogrammetry. While a high-resolution photo camera captures a set of images from varying view angles to reconstruct a detailed 3D model of the scene, low-resolution multimodal camera(s) simultaneously record the scene. All cameras are pre-calibrated and rigidly mounted on a rig, i.e., their imaging properties and relative positions are known. The method was realized in a laboratory setup consisting of a professional photo camera, a thermal camera, and a 12-channel multispectral camera. In our experiments, an accuracy better than one pixel was achieved for the data fusion using multimodal superimposition. Finally, application examples of multimodal 3D digitization are demonstrated, and further steps to system realization are discussed.

## 1. Introduction

### 1.1. Motivation

Multimodal imaging has evolved as an essential advancement in computational image analysis and interpretation [1,2,3,4]. Beyond the classical image acquisition by monochrome or color (RGB) cameras, new and/or more comprehensive information is captured by cameras in uncommon spectral regions, such as infrared [5,6,7] and ultraviolet [8,9,10], cameras sensitive to a certain polarization state [11,12,13] or combination of those [14,15,16]. Camera sensors with a mosaic filter array capture multiple modalities for each single snapshot, e.g., multi-/hyperspectral cameras [17,18,19,20]. In our work, we name all cameras of that kind “multimodal camera” for simplicity. In contrast, classical monochrome/color cameras are referred to as “photo camera”.

The fusion of multimodal 2D image data with 3D surface data allows to spatially localize multimodal information on an object surface. Furthermore, information about the object’s shape and pose can improve the outputs of the multimodal data interpretation. Works on the fusion of multimodal 2D image data and 3D surface are found in the areas of criminal investigations, cultural heritage, industry, and medicine.

The VirtoScan project [21,22] aims to digitize forensic subjects, such as injuries or corpses, including 3D surface, 3D volume, and multispectral data. Multimodal data, in combination with shape, reveal new information about cultural heritage objects [23,24,25]. Drones capture multispectral data and topography of the terrain to derive the condition of field crops [26,27] and forest areas [28,29]. In industrial production, fusions of 3D and multimodal data have been investigated for the detection of contaminations [30] and human–robot interaction [31]. Zhang et al. [32] achieved a robust contactless heartbeat measurement with a multimodal camera through the combination with 3D pose data of a subject’s head. That approach allowed for contact-free monitoring of newborn infants’ vital signs [33].

Methods that derive 3D shape data out of a given set of 2D images have both data modalities fused implicitly. Nevertheless, the fusion of 2D photo images and independent 3D shape data is challenging due to the principal differences in their contents [34,35,36]. For multimodal 2D images, the challenge is even greater because their resolution and contrast are typically not equivalent to photo cameras, so prominent features are more difficult to track. Task-specific methods have been developed for the matching and fusion of different multimodal 2D images [37,38,39,40]. Beyond that, their fusion with 3D shape data further increases the level of difficulty.

We demonstrate a solution for the fusion of 2D image data from low-resolution multimodal cameras with dense 3D surface data into multimodal 3D models. Furthermore, our work aims to advise on the implementation of our solution and to highlight its potential for enhancing 3D digitization applications using additional multimodal information, and vice versa, to improve multimodal imaging tasks with 3D shape data.

### 1.2. State of the Art

First, we give an overview of previous works that considered the fusion of 3D surface data with multimodal camera image data.

One popular 3D measurement technique is multi-view photogrammetry (MVP), also named Structure from Motion, abbreviated as SfM [41,42,43]. MVP requires only one single photo camera to obtain 3D models. A scene is photographed from several freely selected viewpoints. The set of photos, together with sophisticated software tools, allows to determine the alignment of the viewpoints, i.e., their 3D poses and the calibration of the camera lens. With this knowledge, a dense 3D point cloud or mesh is reconstructed using the triangulation principle [42]. Finally, the 2D images captured with the photo camera can be fused with the 3D data by using the camera calibration and the poses to project all images as a texture layer onto the surface. If professional digital cameras (DSLRs) are used, high-resolution 3D meshes with photorealistic textures can be achieved [44].

In MVP, the fusion of 3D data with 2D images is implicitly realized using the reconstruction principle. Applying MVP with a multimodal camera is one approach to obtain multimodal 3D models [45,46]. However, MVP relies on a large number of image features in the scene, ideally captured in high resolution and with high contrast, which typical multimodal cameras cannot provide, as they have low spatial resolution (<1 megapixel) and limited contrast for physical reasons. MVP with a single multimodal camera leads to less robustness and quality. The work from Edelman et al. [45] shows that many input photographs are required and that the resolution and quality of the 3D model are, nevertheless, quite low. Our own experiments showed that this method often fails to determine the poses of the viewpoints, achieving no 3D reconstruction at all. Chane et al. [25] created a complex setup with photogrammetric markers and an external tracking camera to measure the viewpoints of the multimodal camera.

A known approach to improve MVP with multimodal images is the combination of photo images of the same scene. The MVP workflow is started based on the photo camera images. With the photo camera poses known, the poses of the multimodal images need to be derived to incorporate them into the MVP workflow. Two kinds of methods are found in the literature: (1) linkage between photo and multimodal images by feature matching and (2) using the same physical camera unit during image acquisition for photo and multimodal images.

Customized feature descriptors were developed for the matching between multimodal and photo images [37,38,39,40,47,48]. This matching is hard to automate for many applications so that semi-manual procedures are common [49,50]. Found features are used to reproject the multimodal images onto the photo images so that they can be set onto the same viewpoints [47,48,49,50].

When multimodal and photo images are recorded from the same physical camera unit, they are captured sequentially by switching spectral filters in front of the camera [23,24,51,52] and/or changing the illumination source [53]. By using high-resolution monochrome camera sensors, the resolution of the multimodal images itself is increased, leading to better feature detection in general. Nevertheless, feature matching between multimodal images can be omitted because the viewpoints are identical. The camera and illumination setup from Stech et al. [53] is fixed, and the object is moved by a turntable to realize different viewpoints. The main drawbacks of that method are the time-consuming data acquisition and the necessity of a controlled environment. We were looking for an approach using snapshot multimodal cameras that would allow for a quick application with a mobile setup in uncontrolled environments.

Professional multispectral camera units are available for drones [54,55,56]: RedEdge and Altum-PT (AgEagle Aerial Systems Inc., Wichita, KS, USA), Sequoia (Parrot Drone SAS), or P4 Multispectral (DJI). These units contain a set of separate cameras with different spectral sensitivities. Through the separate sensors and their focus on the visible and near-infrared spectrum, the resolution and quality of the images are good compared with mosaic multispectral cameras. Furthermore, the large imaging distance leads to similar fields of vision and, thus, closely matched images. The results of such drone cameras in close-range situations are unknown. We aim for close-range scenarios, i.e., some meters or nearer.

Besides MVP, snapshot 3D sensors exist, such as stereo vision or time-of-flight cameras, which capture a depth map of a scene from a single viewpoint. Heist et al. [57] used multimodal stereo cameras in their setup, which resembles Edelman’s and Aalders’ MVP system [45]. Chen et al. [58] were similar but used filter-wheel cameras. The drawbacks of filter-wheel cameras are analogous to those in the work of Stech et al. [53] in MVP. Nevertheless, for snapshot 3D sensors, an alternative method to fuse 3D shapes with multimodal data has been found. Multiple works [30,31,32,33,59,60,61] have demonstrated setups where a multimodal camera was added to the snapshot 3D sensor. The fusion of the multimodal images with the 3D point cloud was realized by known geometric relations between the 3D sensor and the multimodal camera. Those were determined in a pre-calibration, including intrinsic parameters of the cameras (e.g., distortion) and their relative poses. The main advantage is the omission of any feature matching between the multimodal images and the 3D data. The multimodal images can be projected onto the 3D point cloud using only the known geometric relations.

To conclude our overview of the state of the art, we want to mention that there are special 3D sensors that use multimodal cameras to obtain 3D shape data in the first place where established techniques fail, e.g., for very shiny [62,63,64] or transparent [65] objects or for single-shot scenes [66].

We aimed for a method to fuse 3D and multimodal data dedicated to MVP and not to snapshot 3D sensors. Our novel idea transfers the approach of adding an external multimodal camera from snapshot 3D sensors to MVP-based 3D acquisition systems. A high-resolution photo camera is used as “MVP 3D sensor” and combined with a multimodal camera in a fixed arrangement. The fusion of both data modalities is based solely on pre-calibrated geometric relations between the cameras and no feature matching.

In Section 2, we present our workflow for multimodal MVP and we describe the experimental setup we used to test the workflow. The procedure and results of the pre-calibration are addressed in Section 2.3. Section 3 follows with results of multimodal MVP. We show some exemplary multimodal 3D reconstructions and an evaluation of the accuracy of our data fusion method. Finally, we discuss our results in Section 4, accompanied by a conclusion and an outlook for our method and system setup.

## 2. Materials and Methods

### 2.1. Multimodal Multi-View Photogrammetry

**Figure 1:**
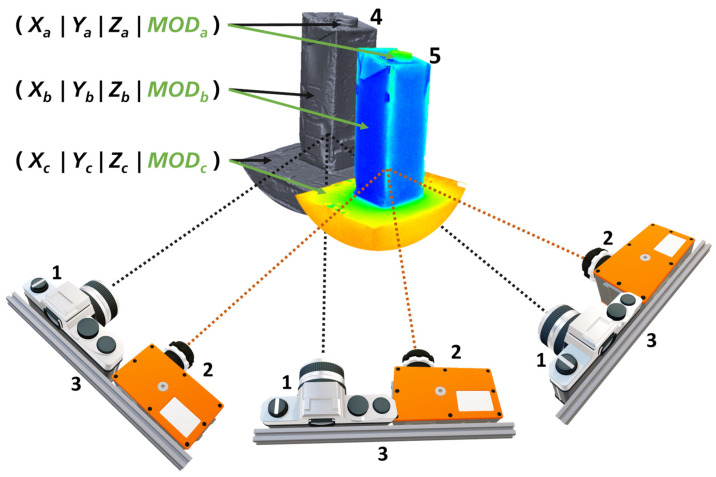
Scheme of our approach for multimodal MVP using one high-resolution photo camera (1) and one multimodal camera (2) in a fixed pre-calibrated arrangement (3). It allows to reconstruct a dense 3D mesh of the scene (4) and fuse it with the multimodal images in form a texture layer (5).

Our main goal was the reconstruction of a high-resolution 3D model by MVP and its fusion with multimodal image data in the form of a texture layer. MVP needs high-resolution photo images from a photo camera to obtain a dense and accurate 3D mesh. Therefore, we propose to combine one or more multimodal cameras, which usually have a low resolution, with a high-resolution photo camera. In our proposed method, all cameras were used in a fixed arrangement, which had been previously calibrated with respect to the camera’s relative external orientations. Our approach of multimodal MVP is shown schematically in Figure 1. The acquisition setup consists of one high-resolution photo camera (1) and one multimodal camera (2) in a fixed arrangement (3). The relative pose between the cameras is known from a pre-calibration procedure. The setup is used to capture a series of images of the scene from different viewpoints. The poses of the viewpoints of the photo camera images (1) are determined in the MVP process and allow to reconstruct a dense 3D mesh of the scene (4). The poses of the viewpoints of the multimodal images are derived from the pre-calibrated relationship to the photo camera so that its image data can be projected onto the 3D mesh as a multimodal texture layer (5). No feature matching between photo and multimodal images is required. Finally, a 3D coordinate (*X*|*Y*|*Z*) and a multimodal value (*MOD*) can be assigned to each surface point. Our approach for multimodal MVP can generally be extended to multiple multimodal cameras so that each surface point can have multiple multimodal values.

**Figure 2:**
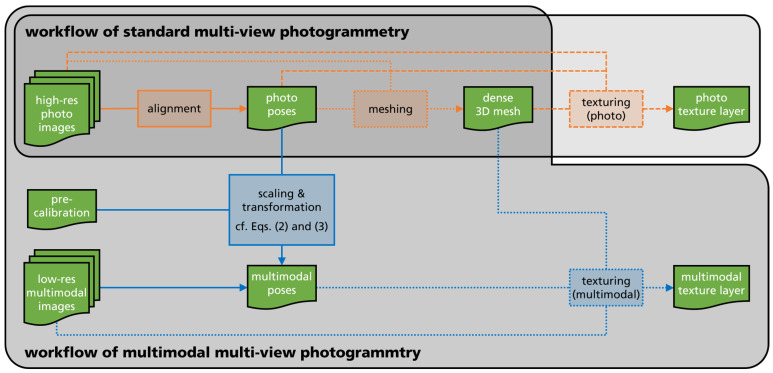
Workflow of multimodal MVP as extension of the standard MVP workflow. The green boxes indicate input data as well as intermediate and final result data. Orange boxes and arrows are the main processing steps of standard MVP, while blue boxes and arrows are extensions by multimodal MVP.

The pre-calibrated camera setup allows for the reconstruction of high-resolution 3D models through MVP on the basis of the photo camera images. The multimodal camera images are projected onto the model in a processing step called “texturing” as a texture layer. Figure 2 shows the workflow of our multimodal MVP and how it extends the standard MVP workflow. The approach can be extended for multiple multimodal cameras as long as at least one high-resolution photo camera is included.

We started with two sets of images: (1) from the photo camera (“high-res photo images” in Figure 2) and (2) multimodal camera (“low-res multimodal images” in Figure 2). It is expected that the sets are built using image pairs captured subsequently from a fixed arrangement, as illustrated in Figure 1. Like with standard MVP, the object of interest was captured from various viewpoints i. The reconstruction of the dense 3D mesh followed the workflow of standard MVP only using the high-res photo images (top row in Figure 2). This consisted of an alignment step to obtain the poses of the photo images and, afterward, the building of the point cloud and mesh.

New processing steps for multimodal MVP are shown as blue boxes and arrows in Figure 2. First, we needed to determine the poses of the low-res multimodal images in relation to the 3D mesh. This was performed by using the poses of the high-res photo images $T_{i,\mathrm{high-res}}$ given from the standard MVP workflow and the pre-calibration $T_{\mathrm{precalib}}$ of the camera arrangement. T is a common representation of a pose in 3D space by a 4 × 4 transform matrix, including a 3 × 3 rotation matrix R and a 3 × 1 column vector t for translation:

(1) $$T=\left[\begin{array}{cc} R & t\\ 0 & 1 \end{array}\right].$$

$T_{\mathrm{precalib}}$ contains the multimodal camera’s pose relative to the photo camera without connection to any exterior object coordinate system. It was determined in a pre-calibration procedure, which we describe closer in Section 2.3, for our experimental setup. $T_{\mathrm{precalib}}$ is constant over the complete image acquisition of a scene.

The alignment step with the photo images in the standard MVP workflow did not produce, indeed, the requested poses $T_{i,\mathrm{high-res}}$. Without prior information, the raw poses $T_{i,\mathrm{high-res}\;\mathrm{raw}}$ are free of scale. Their adjustment to metric scale is a necessary intermediate step before connecting them with $T_{\mathrm{precalib}}$. A specimen with known size or a scale bar in the scene are common methods to define the scale [41,42,43]. Hereby, a scale factor s was determined, which could be applied to the translational vector of $T_{i,\mathrm{high-res}\;\mathrm{raw}}$ to obtain scaled high-res poses $T_{i,\mathrm{high-res}}$:

(2) $$T_{i,\mathrm{high-res}}=\left[\begin{array}{cc} R_{i,\mathrm{high-res}\;raw} & s\cdot t_{i,\mathrm{high-res}\;raw}\\ 0 & 1 \end{array}\right].$$

After that intermediate scaling, the low-res multimodal images poses $T_{i,\mathrm{low-res}}$ could be calculated using the following:

(3) $$T_{i,\mathrm{low-res}}=T_{i,\mathrm{high-res}}\cdot T_{\mathrm{precalib}}.$$

It is emphasized that, hereby, the poses of the multimodal images were determined without any feature matching to the high-res photo images. Our multimodal MVP method is, therefore, not dependent on the characteristics of the scene itself and can even be applied in cases with strongly divergent scene representations between photo and multimodal images.

After the low-res multimodal poses were determined, their image contents can be projected onto the 3D mesh which is taken from standard MVP workflow. In case of multiple multimodal cameras, also multiple texture layers or combination of those could be reconstructed. Of course, the high-res photos could be projected onto the 3D mesh as well to obtain a photo texture layer.

Our method allows for the multimodal 3D digitization of objects using MVP independent to the resolution or image quality of the multimodal camera. The 3D reconstruction itself was realized solely from the high-res images of the photo camera, for which MVP software tools were optimized. The low-res multimodal images had no influence on the quality of the 3D mesh. Their data were projected afterward as texture layer onto the surface.

Furthermore, our method allows for the fusion of multimodal image data taken by distinct cameras. By projecting their image contents onto the object surface, we obtain the full multimodal information for each object point. Existing research work shows [37,38,39,40] that this task is challenging based on the multimodal 2D images itself. Deep learning applications with multimodal data require such methods for early sensor fusion [67].

Our method always requires one high-resolution photo camera, in addition to the multimodal camera(s). Based on our experience, modern photo cameras—even board-level sized RGB cameras—have sufficient quality to apply our method. Their size and price are negligible compared with multimodal cameras.

The major challenge of our method is the pre-calibration between the photo and multimodal camera. It must be realized with a specimen that is rich of contrast in both cameras. Common chessboard or circle targets printed on paper are still applicable at wavelength close to the visible spectrum. In more exotic multimodal channels, special specimen must be created. We show the usage of a board with heatable metal circles. In the ultraviolet spectrum, targets of non-fluorescent paper could be applied [68]. The chessboard corners/circles must be large enough so that they are resolved in the low-res multimodal camera image. The intrinsic calibration (camera constant, principal point, distortion) of the high-res photo camera can be improved making a separate calibration with an adequate specimen.

For the fusion of the multimodal image data and the 3D model, all cameras should be placed as close together as possible to have optimal overlap of their fields of view. Our method requires a rigid alignment of all cameras throughout the data acquisition process, so that $T_{\mathrm{precalib}}$ remains constant. The targeted stability for the camera’s relative position and orientation depends on the concrete application. As a general goal, the resulting deviation of the texture layer should be kept below the size of one multimodal camera pixel to avoid visual impairment (cf. Section 3.3).

### 2.2. Laboratory Setup

**Figure 3:**
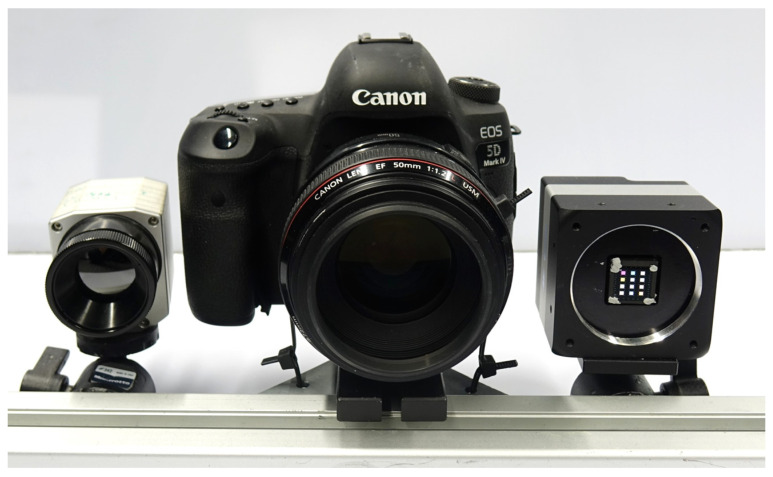
Laboratory setup consisting of a high-resolution DSLR camera (**center**), a 12-channel multispectral camera (**right**), and a thermal camera (**left**).

We tested multimodal MVP, as described in Section 2.1, with a laboratory setup consisting of three cameras. The camera arrangement is shown in Figure 3. The photo camera in the center was a DSLR camera, Canon EOS 5D Mark IV. The first multimodal camera was a thermal camera from Optris with a working range between 0 and 100 °C and a specified accuracy of ±2 °C. The second multimodal camera had a customized multispectral sensor. Here, a monochrome camera from Baumer was equipped with a customized filter and multi-lens array [69], resulting in a multispectral camera with 12 channels of 50 nm bandwidth in steps between 400 and 1000 nm. In our experiments, each channel was treated as a separate multimodal camera. The parameters of the three cameras are summarized in Table 1.

The cameras were mounted on an aluminum beam, which was adaptable to a tripod. The rigidity of the aluminum beam and the camera mounts were sufficient for our purpose, which we checked through the deviation of the multimodal texture layers (cf. Section 3.3). Outside a laboratory environment, the mounting can be realized with stiffer and more temperature-stable materials, such as carbon profiles, to improve the rigidity.

It is useful to keep the distances and angles between the camera small for optimal overlap of photo and multimodal camera images. The objective lenses were selected to have a similar field of view. At a working distance of 0.5 m, an area of approximately 0.5 × 0.35 m^2^ was captured.

In our experiments, we used ceiling fluorescent lights and a desk infrared light bulb with 100 W (IR808 from Efbe-Schott) to obtain reasonable data for all multimodal channels. A Spectralon reflectance standard with >95% diffuse reflectance from 300 to 2000 nm (Zenith Lite from SphereOptics) was used as gray card for white balancing the multispectral camera images.

Our laboratory setup illustrates the large discrepancy between photo and multimodal cameras in terms of pixel resolution. The 30 Mpx of our photo camera was nearly factor 100 higher than the multimodal channels with 0.35 Mpx. Even modern board-level cameras, such as built-in mobile phones, are significantly higher resolved.

### 2.3. Pre-Calibration

The setup of photo and multimodal cameras must be pre-calibrated before applying our multimodal MVP workflow. In particular, the relative external orientation between both cameras $T_{precalib}$ was of major interest. Besides the external orientations, intrinsic parameters, according to the pin-hole camera model and distortion, are an outcome of the pre-calibration. Methods for camera pre-calibration are not limited to a single stereo pair but can be extended to multi-camera systems. All geometrical and optical properties can be derived from a set of images of a specimen with prominent unique features. Common methods use planar chessboard, ArUco, or circle patterns [42].

**Figure 4:**
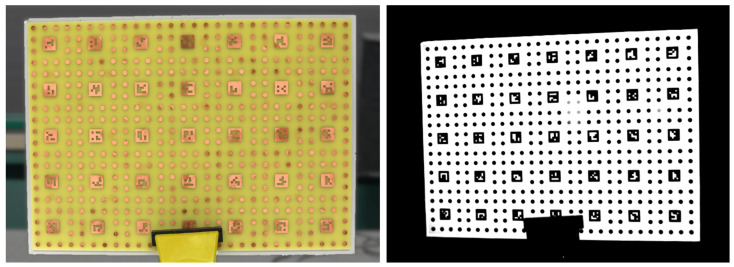
Circle specimen realized as circuit board. It was warmed to 35 °C for pre-calibration between photo (**left**) and thermal camera (**right**). The circles were 5 mm in diameter and 12 mm in distance.

**Figure 5:**
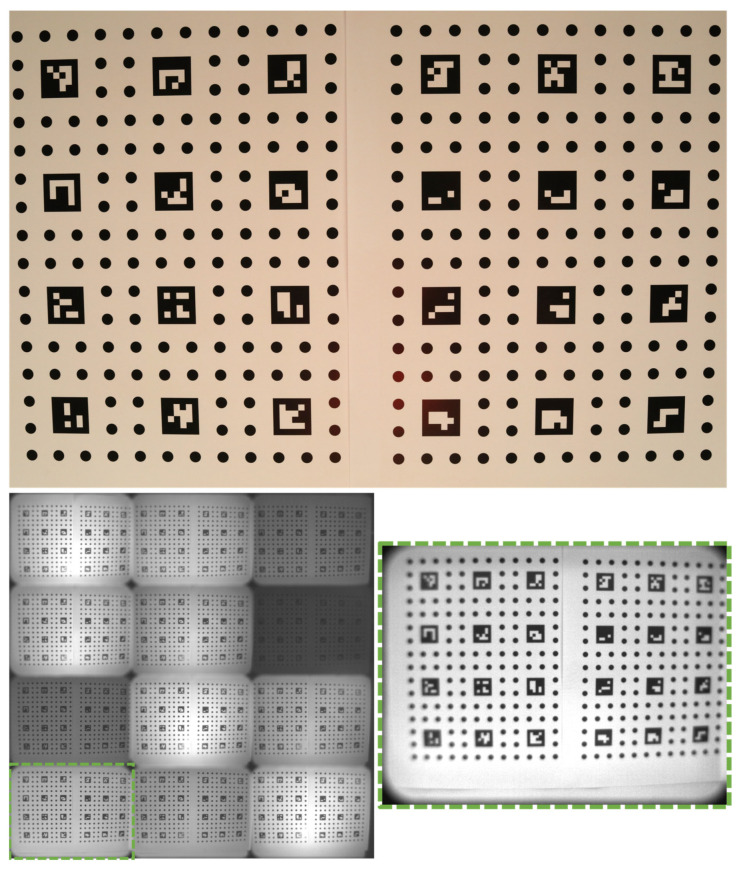
Circle specimen printed on paper for pre-calibration between photo and multispectral camera. Photo image (**top**), full multispectral camera image before splitting the 12 channels (**bottom left**), and split single image of 550 nm channel (**bottom right**) are shown. The circles were 8 mm in diameter and 20 mm in distance.

It must be guaranteed that the features are rich in contrast in all cameras. Because we did not achieve this for all our multimodal cameras at once, we used two different specimens to pre-calibrate our laboratory setup. The customized circuit board in Figure 4 was taken for pre-calibration between the photo and thermal camera. The metallic parts are warmed up to create a thermal contrast. Between the photo and multispectral cameras, the paper printout in Figure 5 was glued on a stiff board. Both specimens contained similar patterns: a grid of circles that are uniquely identifiable by ArUco markers. The size and distance of the features was dictated from the low-resolution multimodal cameras. The heatable pattern consisted of circles with a diameter of 5 mm and pitch distance of 12 mm. In the printout pattern, a diameter of 8 mm and a distance of 20 mm was used due to the larger noise in the images.

In general, our pre-calibration follows state of the art procedures [70,71]:

1. Capture a set of (stereo) images of the specimen in varying orientations;
2. Extract the pixel coordinates of each visible unique feature in each image;
3. Optimize the extrinsic and intrinsic camera parameters by bundle block adjustment;
4. Perform metric scaling of the external orientations using the known pitch distance of the circles.

Multimodal MVP does not require to pre-calibrate all cameras together in case of multiple multimodal cameras. Our method allows for the distinct pre-calibration of each possible stereo pair of photo and multimodal camera in a separate procedure. Pre-calibration 1 covered the photo and thermal camera. Pre-calibration 2 covered the photo camera and the 12 channels of the multispectral camera as separate camera units.

We captured 13 stereo image pairs of the calibration board in Figure 4 for pre-calibration 1. The calibration board in Figure 5 was captured in 16 stereo image pairs for pre-calibration 2. In both cases, the calibration board was placed in various distances between 500 to 800 mm in front of the cameras and tilted in various angles between −25° to +25° relative to the normal of the calibration board.

After stereo image capture, the pixel coordinates of the circle centers were determined in the images by fitting ellipses to their boundaries that consider a perspective distortion of their circular shape. Each circle center was uniquely identifiable between the stereo images through its position to the next neighbored ArUco marker so that image correspondences could be specified. The pixel coordinates of those image correspondences were introduced separately to the bundle block adjustment for pre-calibrations 1 and 2.

The bundle block adjustment was carried out with the software Bingo ATM [72]. The known pitch distances between the circles were thereby introduced to obtain the results in metric scale. Bingo ATM assessed its bundle block optimization with a reprojection error in photo space. We achieved 4.7 µm root mean square (RMS) error in pre-calibration 1 and 2.4 µm in pre-calibration 2.

The outputs of Bingo ATM are the intrinsic and extrinsic parameters of the cameras. The intrinsic parameters could likewise be used in the upcoming multimodal MVP procedure as input value (cf. Section 2.4). The extrinsic parameters were given by Bingo ATM as a 6D vector consisting of three translation values, $t_{x}$, $t_{y}$, and $t_{z}$, and three rotation angles, φ, ω, and κ. For both pre-calibrations 1 and 2, we set the photo camera to the origin so that the 6D vectors of the multimodal cameras equaled their relative orientation. An average 6D vector was then determined across all captured stereo image pairs, i.e., 13 from pre-calibration 1 and 16 from pre-calibration 2. Now, the average 6D vector of each multimodal camera could be reshaped into the 4 × 4 transform matrix $T_{\mathrm{precalib}}$ (cf. Equations (2) and (3)) by:

(4) $$T_{\mathrm{precalib}}=\left[\begin{array}{cc} R_{\varphi }\cdot R_{\omega }\cdot R_{\kappa } & \begin{array}{c} t_{x}\\ t_{y}\\ t_{z} \end{array}\\ 0 & 1 \end{array}\right].$$

Here, $t_{x}$, $t_{y}$, and $t_{z}$ built the 3 × 1 vector for translation t. The rotation matrix R was built by multiplying $R_{\varphi }$, $R_{\omega }$, and $R_{\kappa }$ with the following:

(5a) $$R_{\varphi }=\left[\begin{array}{ccc} cos\;(\varphi ) & 0 & sin\;(\varphi )\\ 0 & 1 & 0\\ -sin\;(\varphi ) & 0 & cos\;(\varphi ) \end{array}\right],$$

(5b) $$R_{\omega }=\left[\begin{array}{ccc} 1 & 0 & 0\\ 0 & cos\;(\omega ) & -sin\;(\omega )\\ 0 & sin\;(\omega ) & cos\;(\omega ) \end{array}\right],$$

(5c) $$R_{\kappa }=\left[\begin{array}{ccc} cos\;(\kappa ) & -sin\;(\kappa ) & 0\\ sin\;(\kappa ) & cos\;(\kappa ) & 0\\ 0 & 0 & 1 \end{array}\right].$$

We obtained for the stereo camera setup consisting of photo and thermal cameras (pre-calibration 1) the following 6D vector: $t_{x}=114.72\;mm$, $t_{y}=14.06\;mm$, $t_{z}=-6.86\;mm$, φ=−8.01°, ω=0.56°, and κ=0.45°, resulting in the following:

$$T_{\mathrm{precalib},\mathrm{thermal}}=\left[\begin{array}{cccc} 0.9902 & -0.0091 & -0.1393 & 114.72\\ 0.0078 & \;0.9999 & -0.0098 & \;\;14.06\\ 0.1394 & \;0.0086 & \;0.9902 & -6.86\\ 0 & 0 & 0 & 1 \end{array}\right].$$

It is pointed out that the translation is related to the distance of the camera centers of the modeled pin-hole cameras and not the physical distance (cf. Table 1). In the camera setup consisting of photo and multispectral camera, one $T_{\mathrm{precalib}}$ for each of the 12 spectral channels was determined. Notably, we obtained for the 550 nm channel the following:$\;t_{x}=-81.62\;mm$, $t_{y}=6.69\;mm$, $t_{z}=19.19\;mm$, φ=4.83°, ω=−1.25°, and κ=−0.10°, resulting in the following:

$$T_{\mathrm{precalib},550\mathrm{nm}}=\left[\begin{array}{cccc} \;0.9964 & -0.0000 & \;0.0842 & -81.62\\ -0.0018 & \;0.9998 & \;0.0218 & \;6.69\\ -0.0842 & -0.0219 & \;0.9962 & \;19.19\\ 0 & 0 & 0 & 1 \end{array}\right].$$

In our bundle block adjustment, we also determined intrinsic parameters, together with the extrinsic ones for our camera setups. Nevertheless, it is possible to enter intrinsic parameters as prior to the processing and optimize only the extrinsic parameters. The high-resolution photo camera could be calibrated intrinsically more accurately with a denser feature pattern prior to our pre-calibration. Furthermore, the angle coverage of ±25° in the acquisition of the calibration board could be increased to improve the accuracy of the bundle block adjustment. However, our experiments did not give us reason to use an extended pre-calibration procedure.

### 2.4. Implementation of the Multimodal MVP Workflow

After the pre-calibration of our laboratory setup (cf. Figure 3), we were able to apply multimodal MVP following the workflow in Figure 2. We created an automated script for quick, synchronous image acquisition from all three cameras in our setup. Especially for scenes with thermal content, a quick acquisition procedure was important to keep interim temperature changes small. We also conducted experiments using only one of the two multimodal cameras, depending on the content of the scene.

Images from the photo camera were saved in JPG format. The images from the thermal camera were saved as float arrays, including the raw temperature values in degrees Celsius. For further processing, we selected an appropriate temperature range and converted the data into 8-bit image files in TIFF format. The raw images from the multispectral camera were split with respect to the sensor grid into 12 distinct channels. Each channel was treated as an independent camera. White balancing was applied to the multispectral images. This was derived from a reference acquisition of the Spectralon standard under the same environmental illumination.

All captured images were saved in one directory with an appropriate naming convention. Together with the pre-calibrations $T_{\mathrm{precalib}}$ for each multimodal camera, our multimodal MVP processing could be applied. Moreover, the intrinsic camera parameters (camera constant, principal point, distortion) determined in the pre-calibration were introduced prior to multimodal MVP. We used the photogrammetry software Agisoft Metashape (version 2.0.2) and its Python programming interface (API) to implement the process steps in Figure 2. In one intermediate step, the raw poses of the photo images $T_{i,\mathrm{high-res}\;\mathrm{raw}}$ were scaled after the alignment step from Metashape. In our implementation, we used a scale bar next to the measurement object within the scene. The scale bar consisted of two circles with a center distance of 30.982 mm, which was measured using the 3D scanner HandySCAN BLACK Elite from Creaform with a certainty of ±0.012 mm. The 3D positions of the two circle centers were determined using the intersection from their subpixel coordinates in the photo images. The relation between the reference and the measured distance gives the scale factor s, which was applied to $T_{i,\mathrm{high-res}\;\mathrm{raw}}$, according to Equation (2). The final results of the whole multimodal MVP workflow were a dense 3D mesh and the poses of all captured viewpoints, including the multimodal cameras $T_{i,\mathrm{low-res}}$. With Metashape, we were able to select images belonging to a certain multimodal channel and project texture layers of interest onto the 3D mesh.

Metashape includes its own tool for handling rigid multi-camera systems called “Camera-Rig”, which can help to realize some of the process steps of multimodal MVP. In our context, the photo camera was set as master, while the multimodal cameras were slaves. Pre-calibrated external orientations were entered as prior in the form of the 6D vectors (three translations and three rotations) obtained using Bingo ATM (cf. Section 2.3). However, we observed some artifacts and mismatches in the created multimodal texture layers. We concluded that the “Camera-Rig” tool expects to find some feature matches between the different cameras and misfunctions otherwise. Therefore, we used our own Python implementation for the multimodal MVP workflow, which does not require any feature matching between the images of photo and multimodal cameras.

## 3. Results

### 3.1. Experiments Applying Multimodal MVP

**Figure 6:**
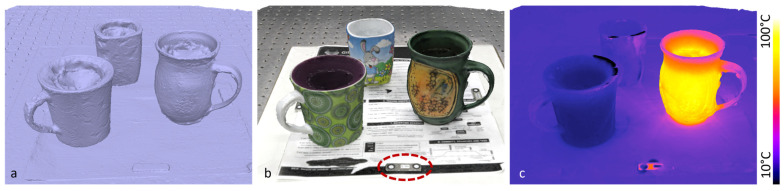
Results of multimodal MVP from the arrangement of three cups with contents at different temperatures. The raw 3D mesh (**a**) is shown with the RGB texture (**b**) and the thermal characteristic (**c**).

In order to evaluate our multimodal MVP method, we used the setup described in Section 2 and assembled a set of scenes. In the first example, we captured an arrangement of three cups with water at different temperatures. We recorded the scene from 18 viewpoints in one hemisphere with the photo and the thermal camera. The 3D reconstruction is shown in Figure 6 as raw 3D mesh (a), mesh with RGB color layer (b), and mesh with temperature as false color layer (c). The cups contained one fridge-cold fluid (left cup), one room-warm fluid (background cup), and a boiling-hot fluid (right cup). The scale bar was placed in the front of the scene (dotted ellipse in Figure 6b). We determined a scale factor of s=210.998 and applied it to the raw poses $T_{i,\mathrm{high-res}\;\mathrm{raw}}$ of the photo images, according to Equation (2).

The complete acquisition procedure took about 10 min, with minor temperature changes in between. The complete acquisition time was mainly determined using the manual repositioning of the camera setup. The hot cup cooled down by ~17 °C, and the cold cup warmed up by ~1 °C between the start and end of the acquisition procedure. Temperature changes during image acquisition would not disturb the reconstruction of the 3D mesh, as this is performed solely on the basis of the photo images (cf. Figure 2). In the multimodal texture layer, an approximate average temperature is usually determined from all multimodal images. However, there are also texturing methods that use the maximum (or minimum) value. The 3D reconstruction took about 3 min on a laptop with Intel Core i9-9980HK CPU and Nvidia GeForce GTX 1650 GPU.

**Figure 7:**
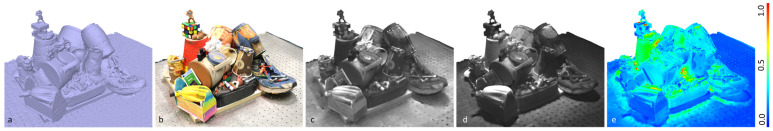
Results of multimodal MVP of a recreated scrap pile. The raw 3D mesh (**a**) is shown with the color texture (**b**), its spectral characteristics at 550 nm (**c**) and 750 nm (**d**), and the ratio between those in false colors (**e**).

In the next example, we applied multimodal MVP to a recreated scrap pile. It consists of multiple things varying in size, material, and color in a chaotic compilation. We captured images from 17 viewpoints in one hemisphere with the photo and multispectral camera. We determined a scale factor of s=238.736 on that scene. Finally, we calculated color layers for each of the 12 spectral channels or combinations of those. Exemplary layers are shown in Figure 7.

**Figure 8:**
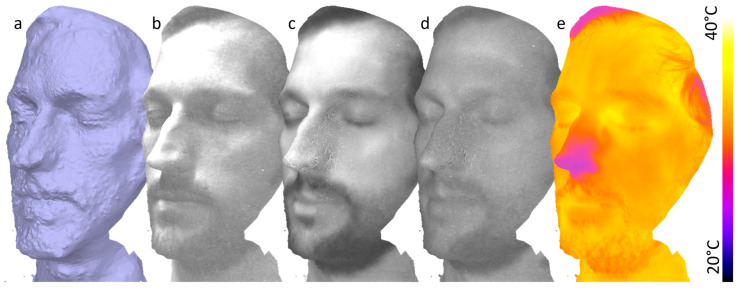
Result of multimodal MVP of a human head. The raw 3D model (**a**) is shown with the spectral characteristic at 450 nm (**b**), 700 nm (**c**), 950 nm (**d**), and the temperature characteristic (**e**).

In the next experiment, we used the multispectral as well as the thermal camera to capture multimodal characteristics of a human head. We captured seven viewpoints in about 2 min. The scale bar was placed on the shoulder of the proband. We determined a scale factor of s=286.074 on that scene. Due to slight motions of the proband, fast data acquisition was essential. Still, the mesh is noisier than the static examples above. The 3D mesh and exemplary multimodal texture layers are shown in Figure 8.

The results on the human head demonstrate how multimodal MVP allow the overlay of multimodal data arising from different camera units. For each 3D point on the surface, we obtain the spectral and temperature characteristics, which enable new properties of the object to be explored.

### 3.2. Comparison of Multimodal MVP against Standard MVP

**Figure 9:**
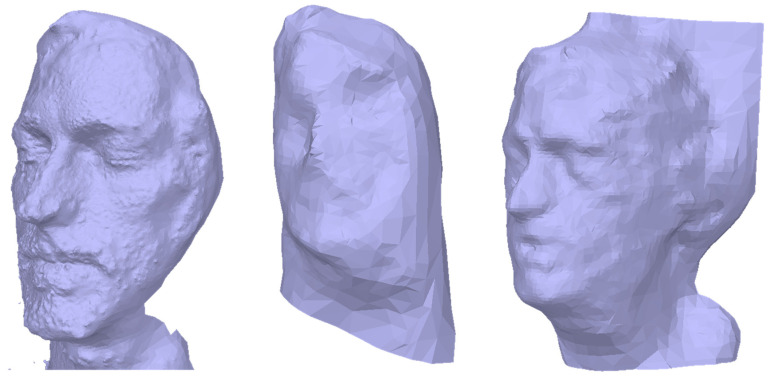
Results of multimodal MVP (**left**), MVP based on the multispectral channel 700 nm (**center**), and thermal images (**right**) for a human head.

We verified the advantages of our multimodal MVP workflow by comparing it against the standard MVP workflow with a single camera, e.g., used by [45,46]. Figure 9 compares the results of multimodal MVP against standard MVP using only the images of one of the multimodal cameras in the example of the human head.

Left is the 3D mesh achieved by our multimodal MVP workflow. In the center, the images of the 700 nm channel from the multispectral camera were used. These showed the highest contrast among all channels of the multispectral camera. The reconstructed 3D mesh shows no familiarity with a human face. Right is the 3D mesh reconstructed only from the thermal camera images. Here, the quality allows for the recognition of a human face, but the detail level is still smaller than our multimodal MVP approach and includes severe artifacts on the backside of the head. A quantitative comparison of the meshes is, therefore, not reasonable. We also tested standard MVP to the remaining examples in Section 3.1. However, none of these instances allowed for the reconstruction of a 3D mesh, as the multimodal images lacked prominent image features.

### 3.3. Accuracy of Multimodal MVP

Our experiments in Section 3.1 demonstrated the capability of our multimodal MVP method to reconstruct different multispectral texture layers on a dense 3D mesh. However, it was not possible to evaluate quantitively the error of the texture projection based on the shown examples apart from the fact that no mismatch was observed visually. For a more sophisticated statement about the projection accuracy of multimodal MVP, we used the specimen used at the pre-calibration of our camera setup (cf. Figure 4 and Figure 5).

We captured new sets of images from both specimens. Then, the multimodal MVP workflow was applied to the image sets according to the experiments in Section 3.1 without any circle or ArUco marker detection. The scale bar was placed in each case beside the specimen. We determined scale factors of s=259.628 for the circuit board specimen (cf. Figure 4) and s=369.426 for the specimen printed on paper (cf. Figure 5). Only after the reconstruction of the 3D mesh and multimodal texture layer was finished, the circle positions were used to evaluate the error of the texture projection. The position of each circle center in 3D space was calculated from its pixel coordinates in the camera images using forward intersection in space based on the viewpoints of those images. References of those positions were determined through forward intersection from only the photo camera images. The same was repeated from only the multimodal camera images. The error of multimodal texture projection was determined as average spatial deviation from the reference positions.

**Figure 10:**
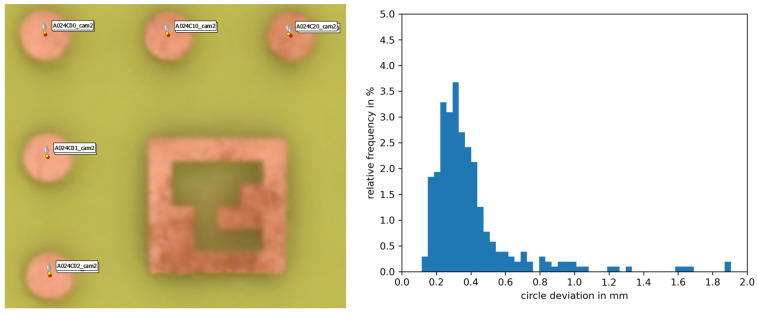
Accuracy of the multimodal texture projection for the setup of photo and thermal cameras. (**Left**): A small section of the specimen showing the reference 3D positions of the circle centers in red and the positions in the multimodal texture layer in yellow. (**Right**): Histogram distribution of the circle deviations.

Figure 10 shows the results for the photo and thermal cameras. Left is a small section of the 3D mesh with RGB texture layer. The determined positions of the reference circles from the photo camera images are depicted in red, whereas those from the thermal camera images are represented in yellow. The right histogram shows the distribution of deviations for all visible circles. We obtained an average deviation of 0.4 mm from the reference positions. This equals about 0.5 pixels of the thermal camera.

**Figure 11:**
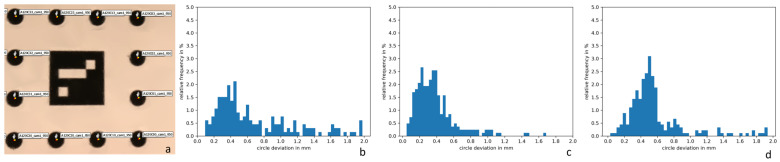
Accuracy of the multimodal texture projection for the setup of photo and exemplary channels of the multispectral camera. Left (**a**): A small section of the specimen shows the reference 3D positions of the circle centers in red and the positions in the multimodal texture layer for channel 950 nm in yellow. Right (**b**–**d**): Histogram distributions of the circle deviations for channels 450 (**b**), 750 (**c**), and 950 nm (**d**).

Figure 11 shows the same evaluation for the setup of photo and multispectral cameras. In principle, all 12 channels could have different errors because they were treated as separate camera units. The circles deviated in average between 0.38 mm for the 750 nm channel, 0.57 mm for the 950 nm channel, and 0.68 mm for the 450 nm channel, corresponding to ~0.5–0.8 pixels in the multispectral camera.

A closer look at the deviations showed that there is a systematic error in the form of a preferential direction. This error pattern indicates a residual error in the scaling during multimodal MVP or small changes in the camera setup after pre-calibration due to limited mechanical robustness. A larger scale bar may improve the accuracy as well. Further measurements of the deviations at different distances or on a non-planar target could make it possible to identify systematic distance-dependent errors, e.g., due to biases in the pre-calibrated focal lengths. However, the residual mismatch of below 1 px was sufficient to avoid any visual artifacts in the multimodal texture layers.

## 4. Discussion and Conclusions

We demonstrated a workflow enabling the fusion of multimodal 2D images with 3D surface data. The main idea of our workflow is the combination of the multimodal camera(s) with a high-resolution photo camera in a fixed setup (cf. Figure 1). The MVP principle allows to obtain high-quality 3D meshes from the photo images. Through the pre-calibrated geometric relation between the cameras, we could obtain the viewpoints of the multimodal images in relation to the photos and the 3D mesh. No image feature matching was required for that connection of the sensors. The fusion of the multimodal 2D image data with the 3D surface data was finally realized by projecting them as a texture layer onto the mesh. We achieved an accuracy of 0.4 to 0.68 mm for the projection of the multimodal images to the mesh in our experiments, which equaled less than a 0.8-pixel mismatch. We traced a major part of this residual error back to limitations in the mechanical robustness of our setup or to the scaling procedure within the workflow because a systematic offset can be observed. However, that error was small enough not to disrupt the visual quality of the fused data.

A major benefit of our multimodal MVP method is the combination of multimodal images with a high-resolution 3D mesh. The low resolution and low image quality of typical multimodal snapshot cameras do not allow to reconstruct any recognizable 3D mesh at all or in low quality and resolution (cf. Figure 9). Our method also obtains the viewpoints of the multimodal images. Through the possibility of expanding our workflow to multiple multimodal cameras, the diverse information they provide can be fused at each surface point. We demonstrated this in Figure 8 by fusing the thermal and spectral data for the 3D mesh of a human head. This aspect of our method could be applied to early sensor fusion in deep learning applications [67].

An advantage of MVP, in general, is its scalability in terms of field of vision. There are system realizations for digitizing small objects, such as insects [73], up to large ones, such as buildings and landscapes [28]. This advantage holds true for our multimodal MVP method as well, making it suitable for many applications.

The experimental scenarios shown in Section 3 address some potential practical applications for multimodal MVP on a small scale. The detection and recognition of the material of an object, together with its shape and position, is useful for automation in the recycling industry. Scrap piles could be digitized using autonomous vehicles, which could directly collect objects of a certain material sort detected through their spectral fingerprint [74]. The examination of our method for the field of crime scene investigations holds promise. The estimation of time since death [75] or age of blood stains [76] is based on thermal and multispectral data that could benefit from the combination with 3D shape data, as demonstrated in Figure 8. Our method has the potential to bring the ideas of the VirtoScan project [21,22] from the lab environment to crime scenes. Another interesting connection can be made with physically based rendering techniques [77], which add material and surface characteristics to 3D models for their realistic presentation. For example, this is a desired feature in upcoming E-commerce environments where retail websites provide interactive 3D models. Methods for classifying materials or estimating surface roughness based on multimodal data, such as polarization and spectral reflection, are found in the literature [78,79,80]. Multimodal MVP could allow an automatic evaluation of those characteristics in 3D models captured using photogrammetry in order to obtain a realistic 3D rendering. More potential applications of the multimodal MVP method can be identified in the literature (cf. Section 1).

Our experiments showed the performance of the presented multimodal MVP method for a multispectral and a thermal camera. Nevertheless, the principle is applicable to other kinds of multimodal cameras, such as ultraviolet or polarization-sensitive cameras as well. The application to more exotic cameras like photon-counting (SPAD) [81] or acoustic cameras [82,83] would need further experimental research.

Advancing our multimodal MVP method regarding its usability in real-world scenarios is the crucial next step. A drawback of our method is the necessity of an additional high-resolution photo camera beside the multimodal camera. In our experiments, we used a professional DSLR camera, which led to drawbacks in terms of overall sensor size, weight, and price. Nevertheless, MVP is widely applied to small industrial [84] and board-level cameras (e.g., smartphones, drones). Those miniaturized photo cameras already clearly outperform typical multimodal cameras in terms of resolution, which is of major relevance to the application of our method. Small-sized and lightweight sensor setups, which could be used handheld or attached to autonomous robot vehicles, are feasible. The cost factor of the additional photo camera would be negligible compared with a multimodal camera. Another point regarding usability is the pre-calibration procedure, which needs to be simplified to allow flexible configuration for different multimodal cameras and quick performance at the operation size. Calibration methods that require only a single shot of a specimen exist and must be tested in terms of robustness and accuracy for our multimodal MVP method (e.g., [85] or [86]).

In conclusion, multimodal MVP has great potential to enhance existing 3D digitization results by adding multimodal information. Conversely, recent multimodal imaging tasks could gain from adding 3D shape data as a further modality.

## 5. Patents

The work reported in this manuscript is registered for a patent under the number DE102021203812B4.

## Figures and Tables

**Table 1:** Parameters of the three cameras in our laboratory setup.

	Photo Camera	Multispectral Camera	Thermal Camera
camera type	Canon EOS 5D Mark IV	Baumer LXG-40MS	Optris PI640
resolution	6720 × 4480 pixel	612 × 582 pixel per channel	640 × 480 pixel
pixel size	4.3 µm	5.5 µm	17 µm
range	16-bit RGB	8-bit	0 °C–100 °C
lens type	Canon EF 50 mm F/1.2 L USM	micro-lens array	IR lens
focal length	50 mm	5 mm	18.4 mm
exposure time	20–200 ms	40–120 ms	~30 ms
distance to photo camera	–	~90 mm	~135 mm
angle to photo camera	–	~2.3°	~2.1°

## Data Availability

Data underlying the results presented in this paper are not publicly available at this time but may be obtained from the authors upon reasonable request.
